# Antibacterial responses of retinal Müller glia: production of antimicrobial peptides, oxidative burst and phagocytosis

**DOI:** 10.1186/1742-2094-11-33

**Published:** 2014-02-18

**Authors:** Pawan Kumar Singh, Melissa J Shiha, Ashok Kumar

**Affiliations:** 1 Department of Ophthalmology/Kresge Eye Institute, Wayne State University School of Medicine, 4717 St. Antoine, Detroit, MI 48201, USA; 2 Department of Anatomy and Cell Biology, Wayne State University, Detroit, MI, USA

**Keywords:** Müller glia, Antimicrobial Peptides, ROS, RNS, Phagocytosis, *S. aureus*

## Abstract

**Background:**

We have previously shown that, in response to microbial infection, activated Müller glia secrete inflammatory cytokines/chemokines and exhibit antimicrobial properties. The aim of this study is to understand the mechanisms and the key components involved in this response.

**Methods:**

Immortalized human retinal Müller glia (MIO-M1 cells) were challenged with *Staphylococcus* (*S*) *aureus*, the leading cause of severe intraocular infection followed by RT^2^ profile PCR array analysis. The expression of human β-defensin 1 (HBD1), 2 (HBD2), 3 (HBD3), hepcidine and cathelicidin LL37 was checked by RT-PCR and quantified by Taqman® qPCR. The expression of AMPs was confirmed at protein level by dot-blot analysis. The production of ROS was measured by dicholoro-dihydro-fluorescein diacetate (DCFH-DA) staining by flow cytometry as well as fluorescence microscopy. The level of nitric oxide (NO) was measured by measuring a stable metabolite, nitrite using the Griess reagent. *In vitro* killing assay was performed by Live/Dead® *Bac*Light™ staining as well as by dilution plating in suspension and adherent conditions following *S. aureus* infection. Phagocytosis was measured by CFU enumeration following infection.

**Results:**

PCR array data showed that, in comparison to uninfected control cells, bacterial challenge significantly (> two-fold) induced the expression of 26 genes involved in cytokine/chemokine, antimicrobials, Toll-like receptor, apoptotic, and NF-κB signaling. RT-PCR analysis showed time-dependent increased expression of HBD1, HBD2, HBD3, LL-37, and hepcidin mRNA in bacteria-challenged Müller glia. The expression of these antimicrobial molecules was also increased at the protein level in the culture supernatant, as detected by dot-blot analysis. Additionally, the bacteria-stimulated Müller glia were found to produce reactive oxygen (ROS) and reactive nitrogen (RNS) species. *In vitro*, killing assays revealed that Müller glia exhibited bactericidal activity against *S. aureus* in both adherent and suspension cultures. Furthermore, our data demonstrated that Müller glia can phagocytize and kill the bacteria in a time-dependent manner.

**Conclusions:**

These data suggest that retinal Müller glia behave like classical innate immune cells by producing a variety of antimicrobial molecules in response to bacterial challenge, suggesting their pivotal role in retinal innate defense.

## Background

Müller glia are the predominant glial cell type in the retina and have a similar role to astrocytes, oligodendrocytes, and ependymal cells in other regions of the central nervous system (CNS) [1,2]. Müller cells are radial glia that pass through the retina from its inner border to the distal end of the outer nuclear layer and because of their cell processes, they surround neuronal cell bodies, axons and blood vessels [1,2]. Müller cells have many local functions; they stabilize the retinal architecture, provide an orientation scaffold, give structural and metabolic support to retinal neurons and blood vessels and prevent aberrant photoreceptor migration into the sub-retinal space [3,4]. Apart from these important support functions, our recent studies have implicated their role in retinal innate defense against microbial infection, such as bacterial endophthalmitis [5]. Since the overall incidence of bacterial endophthalmitis is relatively low, ranging from 0.016 to 0.46% after ocular surgeries or intravitreal injections [6-11] and up to 17% following ocular trauma [6,12-14], it is intriguing, why Müller cells possess innate defense capabilities against microbial pathogens. First, we previously showed that Müller glia express all known human Toll-like receptors (TLRs), the best-characterized receptors present on innate immune cells and involved in antimicrobial innate defense [15]. Second, they are located strategically, that is their end feet are in inner limiting membrane (ILM), next to the vitreous cavity, the site where bacteria proliferate in endophthalmitis [5]. Third, retinal Müller glia originate from the neuroepithelium (the stem cells of the nervous system) and being a progenitor cell they can divide and differentiate into a number of retinal cell types [16]. Thus, the Müller glia possesses the ability to respond to a variety of infectious and non-infectious stimuli.

Innate defense mechanisms are used by the host to respond to a wide range of microbial pathogens in an acute and conserved fashion. Host cells express pattern recognition receptors (PRRs) that sense pathogen-associated molecular patterns (PAMPs) [17,18]. Following detection, a variety of antimicrobial mechanisms are deployed to kill the pathogen in infected cells and/or tissue. We have previously shown that, in the retina, these early innate defenses are provided by retinal glial cells (Müller and microglia). In response to infectious stimuli retinal glial cells produce and secrete inflammatory cytokines and chemokines via TLR signaling [15,19]. In addition to their role in inflammatory response, TLR activation on innate immune cells leads to the production of antimicrobial peptides (AMPs) [20]. The two best-characterized families of AMPs are defensins [21] and cathelicidins [22]. However, the role of AMPs in retinal innate defense has not been fully investigated.

The retina is protected from microbial infection due to the presence of the blood-retinal barrier (BRB). However, in the case of infectious endophthalmitis, bacteria gain access to the vitreous cavity following ocular trauma or surgery, thus bypassing the BRB [23]. In such situations, the production of AMPs by retinal innate cells may be crucial in limiting intraocular bacterial growth. These AMPs are ubiquitous natural effectors of the host defense system and are conserved in both the plants and animals with both broad-spectrum microbicidal activity and cell signaling functions. The ocular surface tissue has been reported to express β-defensins, hepcidin, cathelicidin and Ribonuclease 7 [24], but the expression of these AMPs in retinal cells has not been studied extensively. Our recent studies have shown the induced expression of one of the AMPs, LL37, in Müller glia in response to *S. aureus* infection [5,25]. Hence, it is reasonable to hypothesize that, in addition to LL37 other AMPs may also be involved in retinal innate defense.

In this study, we used a Superarray to investigate the antibacterial responses of Müller glia challenged with *Staphylococcus* (*S*) *aureus* (SA). We also tested other innate responses such as production of reactive oxygen species (ROS) and reactive nitrogen species (RNS) and the phagocytic activities of Müller glia. Our data suggest that in response to pathogen challenge, Müller glia exhibit the induced expression of AMPs, ROS, and NO. The culture supernatants of activated Müller cells were found to possess bactericidal activity. Further understanding of the antimicrobial mechanisms within the retina will allow us to develop new approaches to prevent intraocular infections.

## Methods

### Cell culture

The immortalized human Müller glia cell line MIO-M1 was maintained in DMEM supplemented with 10% FBS, 1% penicillin-streptomycin and 10 μg/ml L-glutamine. Human embryonic kidney (HEK/293) cells were used as unresponsive control cells and they were also cultured in DMEM with 10% FBS. Whenever needed, cells were grown overnight in serum and antibiotic-free DMEM prior to infection.

### RNA extraction and PCR analysis

Total RNA was extracted from the MIO-M1 cells using TRIzol reagent following the manufacturer’s instruction (Invitrogen, Carlsbad, CA, USA). cDNA was synthesized using 1 μg of total RNA using a Maxima first strand cDNA synthesis kit, as per the manufacturer’s instructions (Thermo Scientific, Rockford, IL, USA). The cDNA was amplified using AMP (HBD1, HBD2, HBD3, LL-37, and hepcidin) gene specific PCR primers. The PCR product and internal control glyceraldehyde 3-phosphate dehydrogenase (GAPDH) were subjected to electrophoresis on 1.5% agarose gel containing 0.5 μg/ml ethidium bromide. Stained gels were captured using a digital camera (EDAS 290 system, Eastman Kodak, Rochester, NY, USA). Real time RT-PCR was conducted in StepOnePlus™ Real-Time PCR system (Applied Biosystems, Grand Island, NY, USA). All primers and Taqman® probes (Prime Time Mini qPCR Assay) were purchased from Integrated DNA technologies (Coralville, IA, USA). The quantification of gene expression was determined via the comparative ΔΔCT method. Expression in the test samples were normalized to the endogenous reference GAPDH level and were reported as x-fold change relative to *GAPDH* gene expression. All assays were performed in triplicate and repeated at least three times.

### PCR array for the antibacterial response genes

A human antibacterial response RT^2^ profile PCR array was performed as per the manufacturer’s instructions (Qiagen, Valencia, CA, USA). Total RNA was extracted from infected MIO-M1 cells and cDNA was prepared as mentioned previously [19]. The cDNA was mixed with RT^2^ qPCR master mix supplied by the manufacturer and real time PCR was performed in a 96-well plate format using StepOnePlus™ Real-Time PCR system (Applied Biosystems, Grand Island, NY, USA). The data were analyzed as per the manufacturer’s recommendation using RT^2^ profile PCR array data analysis templates V4.0.

### Dot-blot analysis

MIO-M1 cells were infected with *S. aureus* for various time periods (2, 4, 8, and 12 hours). PBS treated cells were used as a vehicle control. After incubation, the culture supernatant was collected from each well and centrifuged at 10,000 × g for ten minutes. to remove bacteria and cell debris. The clear culture supernatants were transferred to new tubes for use in the dot-blot assay. The culture supernatants were loaded onto a 0.2 μm nitrocellulose membrane using a BIO-DOT™ apparatus (Bio-Rad, Hercules, CA, USA) and vacuum suction. The membrane was fixed in 10% formaldehyde in Tris buffer saline (TBS) for one hour at room temperature (RT). The membrane was blocked in 5% skim milk made up in TBST (TBS containing 0.05% tween 20) for one hour at RT and incubated with primary antibody for various antimicrobial peptides overnight at 4°C. On the following day, the blot was washed three times in TBST and incubated with respective anti-mouse or anti-rabbit HRP conjugates for one hour at RT. The blot was developed using SuperSignal® West Femto maximum Sensitivity Substrate (Thermo Scientific, Rockford, IL, USA) via chemiluminescence using a Kodak image station 4000R Pro, molecular imaging system (Carestream Health Inc, Rochester, NY, USA). Dot intensity was quantified using Image J analysis software (NIH).

### Measurement of intracellular ROS

ROS production was measured by both flow cytometry and fluorescence microscopy using dicholoro-dihydro-fluorescein diacetate (DCFH-DA). MIO-M1 cells were infected with *S. aureus* (Multiplicity of Infection (MOI) 10:1) and, following incubation for the appropriate time period, cells were collected, washed with cold PBS, re-suspended in PBS containing 10 μM DCFH-DA, and incubated for 30 minutes at 37°C in a CO_2_ incubator. The fluorescence intensity was measured at 488 nm excitation and 525 nm emission using a flow cytometer BD AccuriC6 (BD Biosciences, Ann Arbor, MI, USA). For the fluorescence microscopy, the MIO-M1 cells were grown in a slide chamber and, following infection, cells were washed with PBS and incubated in 50 μM of DCFH-DA for 30 minutes at 37°C. The cells were then washed twice with PBS and observed via fluorescence microscope.

### Nitrite concentration assay

The production of NO was measured indirectly via its stable metabolite nitrite using the Griess reagent, as per manufacturer’s instructions (Cayman Chemical, Ann Arbor, MI, USA). The conditioned growth media from the MIO-M1 cell cultures were added, in sequence, to a 96-well plate. Both the Griess reagent R1 and Griess reagent R2 were then added to the 96-well plate. The 96-well plates were allowed to develop for ten minutes at RT, and the absorbance was measured using a plate reader at 540 nm. The nitrite concentration was calculated via comparison to a standard reference.

### *In vitro* bacterial killing assay

Our bacterial killing assay was based on a modification of a method described elsewhere [26-28]. The MIO-M1 cells were washed with Hank’s balanced salt solution (HBSS), and 8 × 10^5^ MIO-M1 cells were co-cultured with 4 × 10^3^ *S. aureus* cells (a 200:1 ratio) in HBSS for one hour at 37°C. Following incubation, the tubes were sonicated, serially diluted, plated onto TSA plates, and incubated overnight at 37°C. The colony-forming units (CFUs) of the co-cultured tubes were compared with the CFUs of growth control tubes containing *S. aureus* only (without MIO-M1 cells).

To estimate bacterial killing by MIO-M1 cells in an adhered condition, MIO-M1 cells were grown in six-well plates and infected with *S. aureus* at a multiplicity of infection (MOI) of 10:1 for four hours. Following incubation, bacterial CFUs were estimated via dilution plating. In the same plate, *S. aureus* growing alone (no cell contact) in DMEM was used as control. Bacterial killing was also estimated using MIO-M1 conditioned medium, deposited on top of *S. aureus* grown on coverslips, via fluorescence microscopy. MIO-M1 cells were infected with *S. aureus* at a multiplicity of infection (MOI) of 10:1 for four hours and the conditioned media were deposited on top of coverslips containing 24-hour growth layers of *S. aureus*. The coverslips were incubated for an additional four hours with after the conditioned medium was added. DMEM medium without any infection was used as a control. Following incubation, the coverslips were stained with the Live/Dead® *Bac*Light™ Bacterial Viability Kit (Invitrogen, Carlsbad, CA, USA), washed three times to remove excess stain and cell debris, and examined under the microscope.

### Phagocytosis assay

MIO-M1 cells (10^6^ cells per well) were grown in small (60 mm) Petri dishes in DMEM medium. The cells were infected with *S. aureus* at a multiplicity of infection (MOI) of 10:1 in each Petri dish and incubated for two hours. Following incubation, the cells were washed and treated with gentamicin (200 μg/ml) for two hours to kill all extracellular and/or adherent bacteria. The absence of extracellular bacteria was confirmed via CFU enumeration on Tryptic Soy Agar (TSA) plates. Two hours after the gentamicin was added, the cells were washed with DMEM and incubated in fresh DMEM containing gentamicin (200 μg/ml) for 2, 4, 8, 12, and 24 hours. For the enumeration of phagocytized bacteria, following incubation the cells were washed three times with PBS and lysed with 0.01% Triton X-100. The lysed cells were scraped and centrifuged at 5,000 × g for five minutes. The cell pellets were washed with PBS and centrifuged at 5,000 × g for five minutes twice more. The pellets were then re-suspended in 1 ml of sterile PBS. Serial dilutions were prepared and the dilutions were plated on TSA plates and incubated overnight at 37°C. The next day, the colonies present on the TSA plates were counted and these counts were expressed in terms of CFUs. Phagocytosis by MIO-M1 cells was also detected by fluorescence microscopy following infection with GFP expressing *S. aureus.*

### Statistical analysis

The Statistical analyses were performed using GraphPad Prism version 6.02 (Graphpad, San Diego, CA, USA). Student’s *t*-test was used for comparison of two groups, whereas one-way analyses of variance (ANOVA) was used for > three groups.

## Results

### *S. aureus* evokes antibacterial gene expression in retinal Müller glia

**Figure 1:**
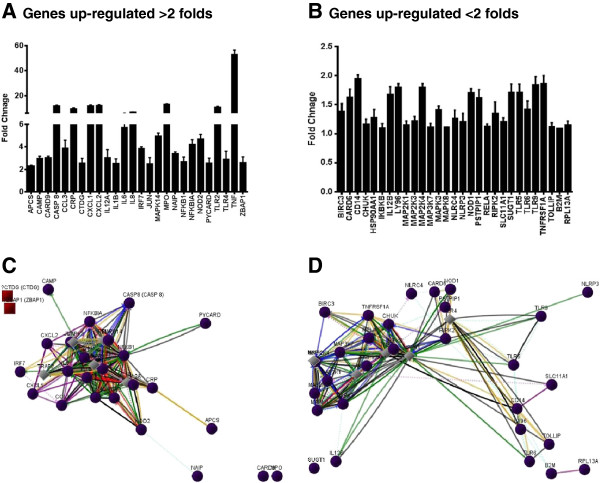
***S. aureus *induces global antibacterial response genes in retinal Müller glia.** The human retinal Müller glia cell line (MIO-M1) was infected with *S. aureus* (SA) for four hours. Total RNA was extracted, reverse transcribed, and subjected to RT^2^ PCR array for human antibacterial response genes. The quantification of induced genes was determined via RT^2^ profile PCR array data analysis software V4 (Qiagen, Valencia, CA, USA). Genes which showed a > two-fold increase in their level of expression are shown in left panel **(A)**, while genes which showed a < two-fold increase are shown in the right panel **(B)**. The data are represented as bar graph and it is a representative of two independent experiments. The interaction among the genes was assessed using central pathway analysis and depicted in panels **(C, D)**. The pathway analysis shows the interaction map of all the upregulated genes following *S. aureus* infection with key central molecules MAPK, NF-κB, and TRAF2. The lines represent: red line, downregulation; green line, upregulation; grey line, regulation; purple line, co-expression; blue line, chemical modifications; orange line, physical interaction; dashed turquoise line, predicted protein interaction; dashed pink line, predicted T-factor regulation; black line, others; grey diamond, neighboring key central molecule; purple circle, genes upregulated in this study; brown square, non-translatable.

In order to determine the innate response of Müller glia towards bacterial pathogens, we performed Human Antibacterial Response RT^2^ Profiler™ PCR arrays. Based on gene selection criteria (*P* < 0.05 and fold change ≥ 2), 26 genes demonstrated upregulation (Figure 1A). Among them, inflammatory mediator genes (IL-6, IL-8, IL-1β, TNF-α, IL-12a, CXCL1, and CXCL2) were predominant, followed by some antimicrobial genes (CAMP and MPO), Toll-like receptors (TLR-2 and TLR-4), NLR and inflammasome signaling genes (NOD2 and PYCARD), apoptotic genes (CARD9, CASP8, and JUN), and some other bacterial pattern recognition receptors (APCS, CRP, and ZBAP1). Additionally, other genes involved in pathogen recognition and initiation of innate responses were also induced in activated Müller glia, but their upregulation was < two-fold (Figure 1B). These genes include TLR-5, -6, and -9, MAP kinases (MAP2K1, MAP2K3, MAP2K4, MAP3K7, MAPK3, and MAPK8), and inflammosomes (NLRP3 and NLRC4). The central pathway analysis shows that the NF-κB, MAPK, and TRAF2 are the key central molecules (hub genes) regulating the expression of multiple effector genes involved in innate and antibacterial responses (Figure 1C and D).

### Müller glia express several antimicrobial peptides in response to *S. aureus* challenge

**Figure 2:**
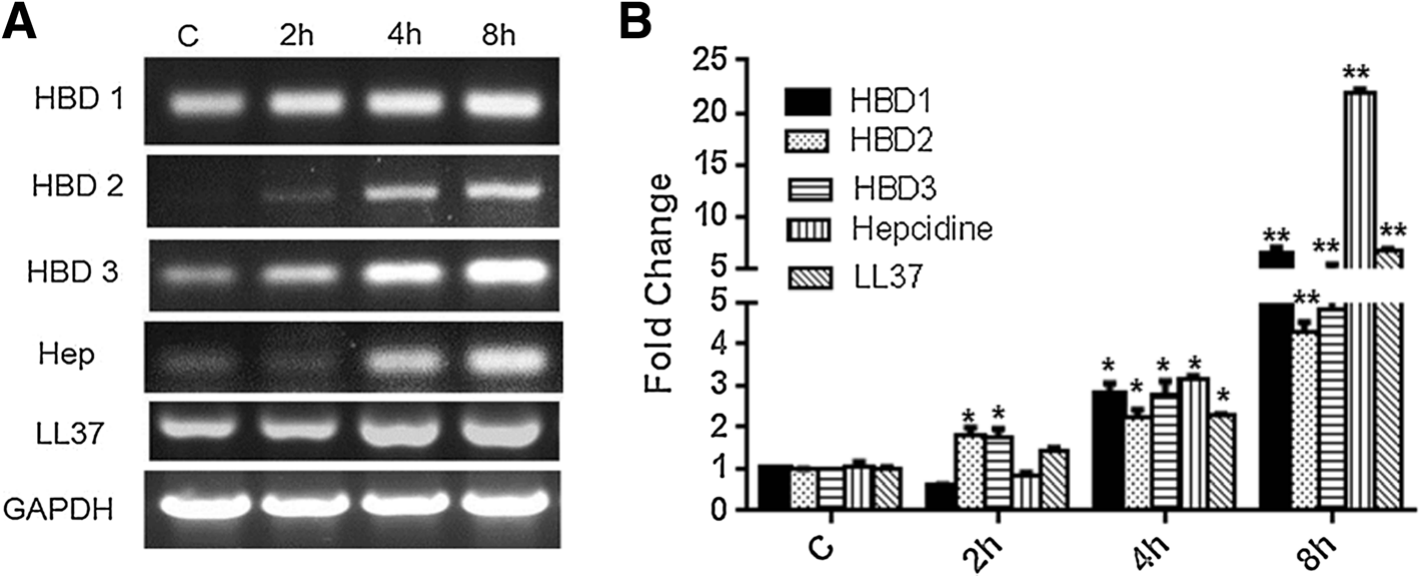
***S. aureus *up-regulates antimicrobial peptides (AMPs) expression in retinal Müller glia.** MIO-M1 cells were infected with *S. aureus* (SA) for the indicated time points. Total RNA was extracted, reverse transcribed, and subjected to semi-quantitative RT-PCR using primers for specific AMPs and glyceraldehyde 3-phosphate dehydrogenase (GAPDH) as the control **(A)**. Real time PCR with a TaqMan® probe was used for the quantification of induced gene expression and the results were expressed as relative fold changes with respect to the GAPDH control **(B)**. Statistical analysis was performed using one-way ANOVA (*P < 0.05; **P < 0.005), for comparisons of control versus stimulated cells over the time. Data points and bars represent mean ± SD of triplicates from three independent experiments.

**Figure 3:**
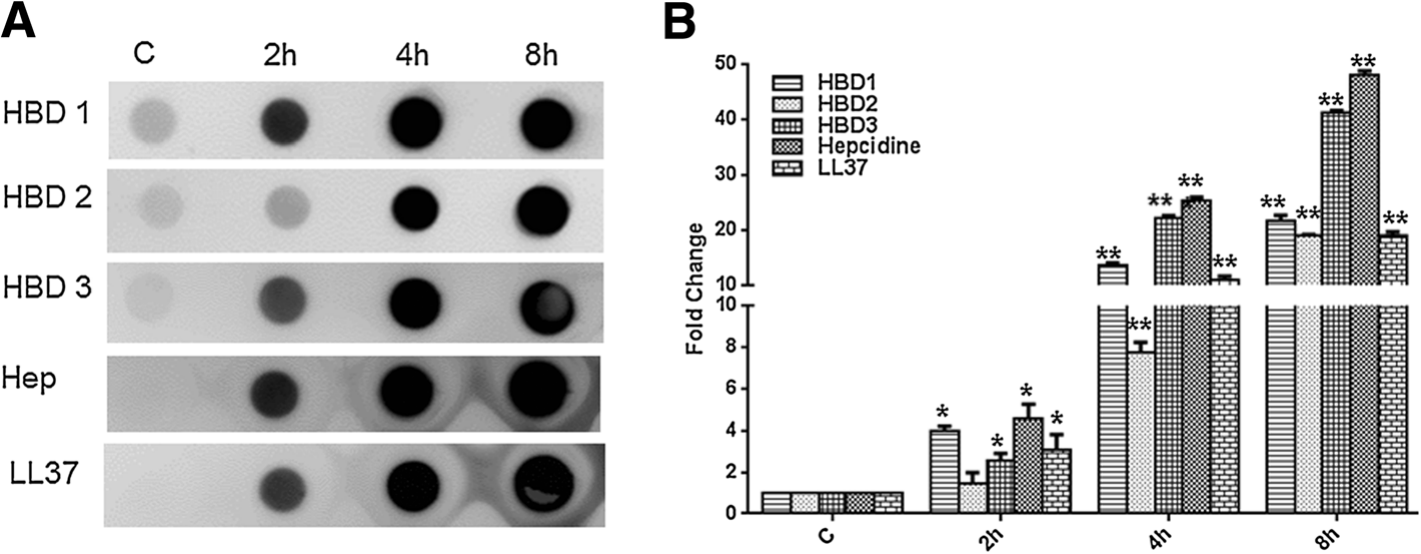
***S. aureus-*activated Müller glia secrete antimicrobial peptides (AMPs) into culture media.** MIO-M1 cells were infected with *S. aureus* (SA) for the indicated time points. The secretion of AMPs into the culture supernatant was detected via dot-blot **(A)**, and the intensities of the dots were quantitated by densitometric analysis and presented as fold-changes, using a value of 1 for the control samples **(B)**. Statistical analysis was performed using one-way ANOVA (*P < 0.05; **P < 0.005), for comparisons of control versus stimulated cells over the time. Data points and bars represent mean ± SD of triplicates from three independent experiments.

We have previously shown that, in response to challenge with *S. aureus*, Müller glia secrete inflammatory cytokines/chemokines and LL37 [29]. In order to investigate whether bacterial stimulation induces the expression of other AMPs such as defensins, time course studies were performed. As shown in Figure 2, *S. aureus* induces the time-dependent expression of HBD1, HBD2, and HBD3, with significant increases at the four hour and eight hour time points (Figure 2A). The expression pattern of another antimicrobial molecule, hepcidin, follows the same trend. As expected, the expression of LL37 was also increased at four hour and eight hour. Concomitant with increased mRNA expression, the activated Müller glia secrete increased levels of HBD1, HBD2, HBD3, hepcidin, and LL37 in culture media, as detected by the dot-blot assay (Figure 3A). Densitometry analysis revealed the time-dependent accumulation of AMPs (Figure 3B). In contrast to Müller glia, no induced expression and secretion of AMPs was observed in control HEK/293 cells following bacterial challenge (data not shown).

### Activated Müller glia exhibit oxidative stress

**Figure 4:**
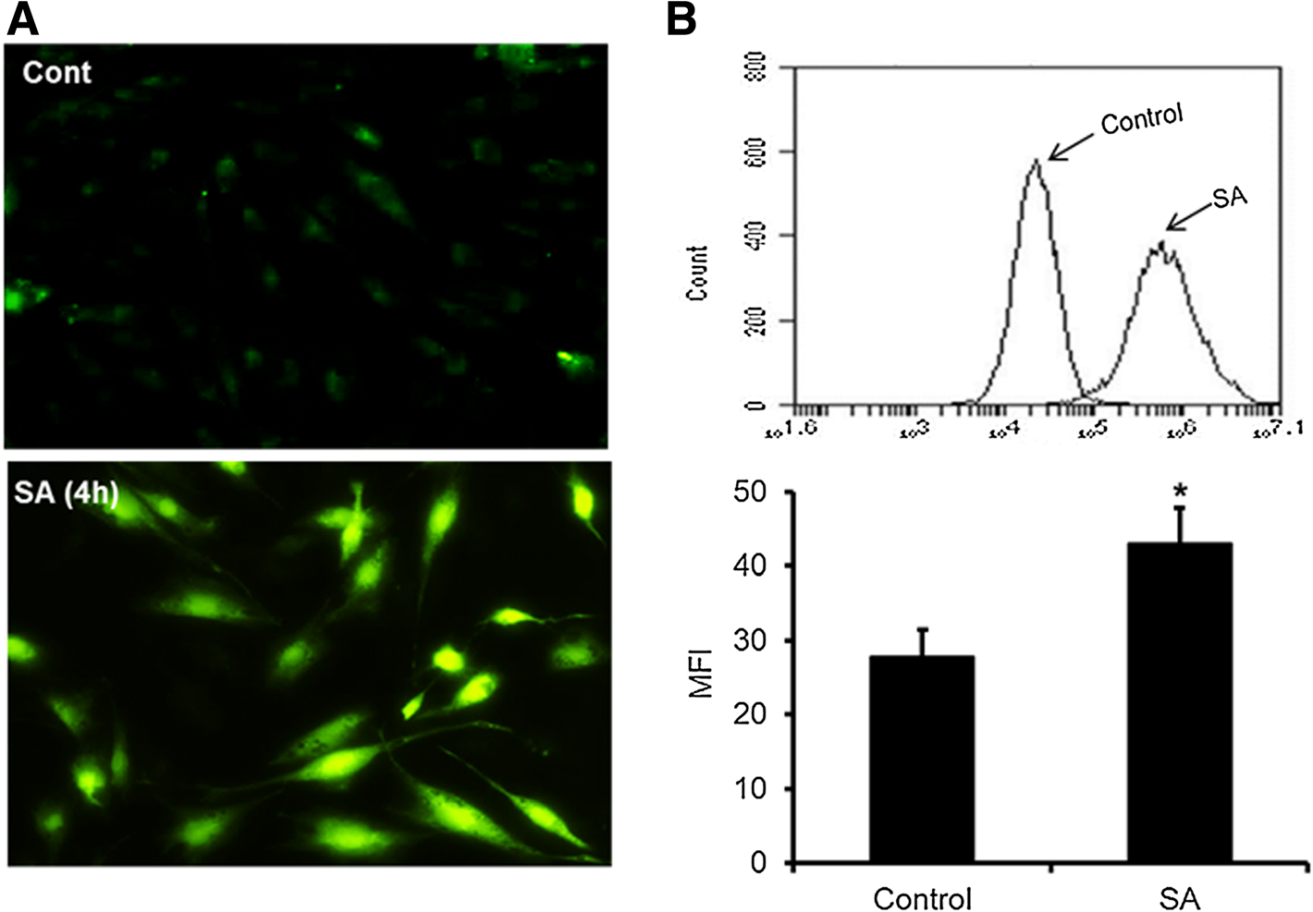
***S. aureus *evokes oxidative stress in Müller glia.** MIO-M1 cells were challenged with *S. aureus* (SA) for four hours. The generation of reactive oxygen species (ROS) was detected via immunostaining **(A)** and ROS quantification was performed using flow cytometry and the data are presented as the mean fluorescent intensity (MFI) after DCFH-DA staining **(B)**. The data represent the mean ± SD of triplicates from three independent experiments. Statistical analysis was performed using a student’s *t*-test for comparison of SA infected cells versus uninfected control cells (*P < 0.05).

**Figure 5:**
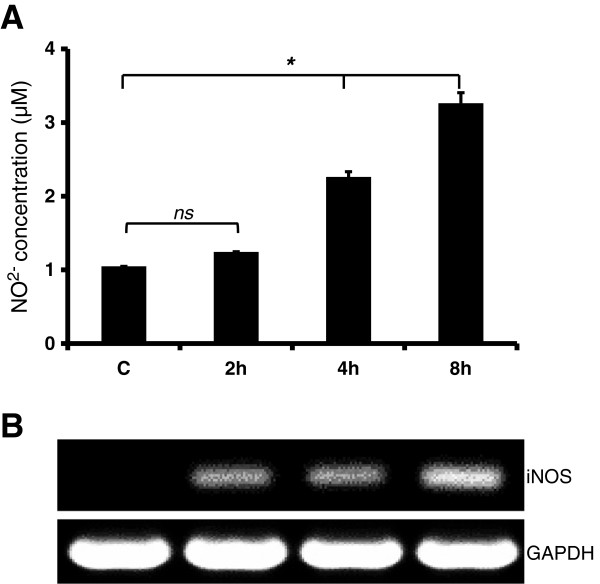
**Müller glia produce reactive nitrogen species following *S. aureus *challenge.** MIO-M1 cells were challenged with *S. aureus* (SA) for the indicated time points. The level of NO in the culture media was determined by measuring the concentration of one of its stable metabolites, nitrite, using the Griess reagent **(A)**. RNA extraction and RT-PCR for inducible nitric oxide synthase (iNOS) expression was performed on the cells. **(B)**. The data represent mean ± SD of triplicates from three independent experiments. Statistical analysis was performed using one-way ANOVA, for comparisons of control versus infected cells over time. (*P < 0.05; ns, not significant).

The oxidative burst, followed by the production of ROS and RNS, is an important component of innate immunity, which assists in the killing of not only the pathogen, but pathogen-infected cells. We investigated the production of ROS and RNS by Müller glia in response to *S. aureus* infection. The immunofluorescence (Figure 4A) and flow cytometry (Figure 4B) assays revealed that Müller glia produced reactive oxygen species (ROS) as a part of an oxidative burst following *S. aureus* infection. The time course studies revealed that ROS generation peaks at four hours with no further increase after this time point (data not shown). In contrast, the levels of NO (indirect measurement of nitrite) were increased in activated Müller glia in a time-dependent manner with the highest detected levels at the eight hour time point (Figure 5A). Consistent with NO production, the mRNA level of inducible nitric oxide synthase (iNOS) is elevated in a time-dependent manner (Figure 5B). Together, these data suggest that Müller cells produce ROS and RNS, implicating their role in antibacterial defense.

### Müller glia-conditioned media possess bacterial killing properties

**Figure 6:**
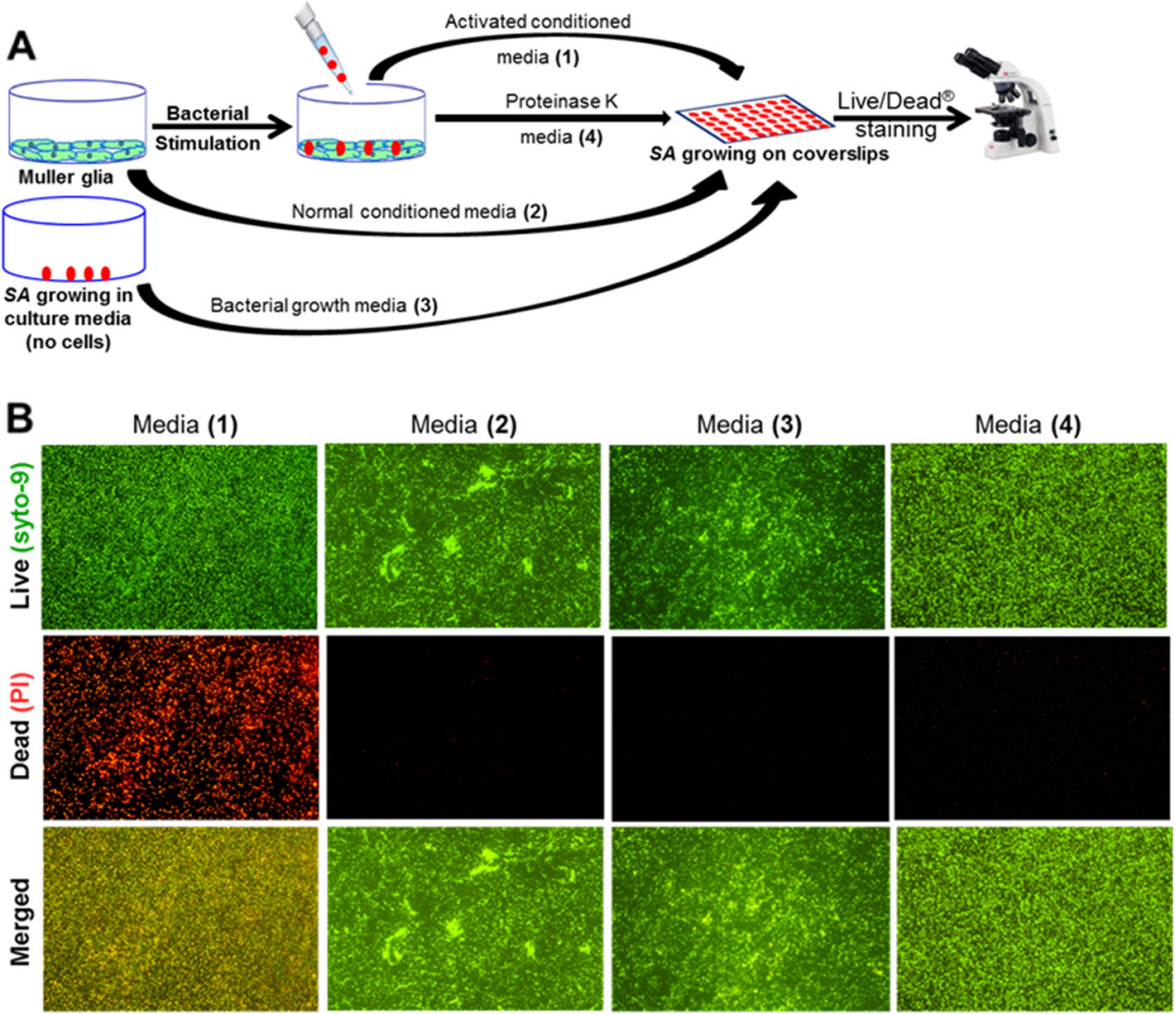
**Activated Müller glia-conditioned media possesses antibacterial properties.** MIO-M1 cells were left either untreated or infected with *S. aureus* (SA) for four hours, the respective ‘normal’ (untreated), ‘activated’ (SA challenged), and proteinase K (20µg/ml for 30 minutes) treated culture media were collected. In a separate set of wells, bacteria were cultured in DMEM media for four hours without cells. All conditioned media were passed through a 0.22 μm syringe filters (bacteria/cell free) and applied to cover slips containing SA biofilms for four hours **(A)**. Bacterial killing was assessed via Live/Dead® staining and subsequent fluorescent microscopy **(B)**. In comparision to untreated, bacterial growth, and proteinase K treated conditioned media, the activated conditioned medium exhibited antibacterial properties, as evidenced by the increased red staining. The experiment was repeated three times independently and the representative Figure is shown.

**Figure 7:**
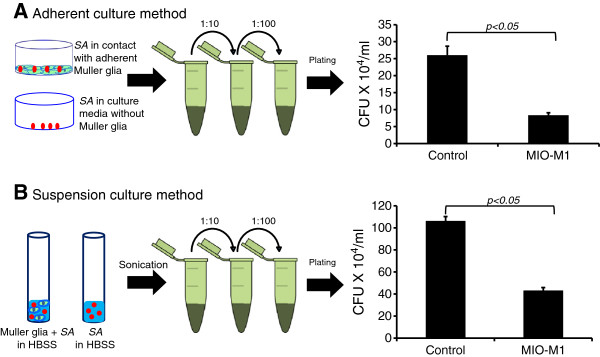
**Activated Müller glia-conditioned media possesses antibacterial properties.** MIO-M1 cells were challenged with *S. aureus* (SA). Following a four hour incubation, the culture supernatant was subjected to a serial dilution and plating for bacterial colony forming unit (CFU) enumeration. Bacteria were also grown in identical culture conditions without Müller cell contact to be used as control **(A)**. In another experiment, suspended MIO-M1 cells (8 x 10^5^) were co-cultured with a 4 x 10^3^ CFU of SA (200:1 ratio) in Hank’s balanced salt solution (HBSS) for one hour at 37°C. The control for this experiment includes bacteria grown separately (without cells). Following incubation, the tubes were sonicated and bacterial CFU was estimated via dilution plating **(B)**. Statistical analysis was performed using a student’s *t-*test (*P < 0.05). The data represent a cumulative of three independent experiments performed in triplicates.

Earlier results indicate that, in response to bacterial challenge, Müller glia produce various antimicrobial molecules. In an attempt to determine the biological functions of these molecules, we performed several bacterial killing assays. As shown in Figure 6, the conditioned medium from the *S. aureus* infected MIO-M1 cells, termed ‘activated’, showed an increased number of dead bacterial cells, as indicated by the intense red staining due to the propidium iodide in the Live/Dead® stain (Invitrogen, Carlsbad, CA, USA). In contrast, the normal conditioned media (media in contact with non-infected MIO-M1 cells) or plain DMEM (no cell contact, just bacterial growth media), when deposited on top of *S. aureus* biofilms, showed heavy green staining due to the Syto9 component of the Live/Dead® stain (Invitrogen, Carlsbad, CA, USA), indicating viable bacteria. Similarly, proteinase K (20 μg/ml for 30 minutes) treatment of the conditioned media showed significantly reduced bactericidal activity, that is increased green fluorescent staining. To further demonstrate the pathogen killing ability of Müller glia, we performed an *in vitro* killing assay in both adhered (Figure 7A) and suspension culture (Figure 7B) of MIO-M1 cells. Both of these assays revealed that Müller glia possess the ability to kill *S. aureus*, as evidenced by the reduced number of viable CFUs when compared to controls.

### Müller glia possess bacterial phagocytic activity

**Figure 8:**
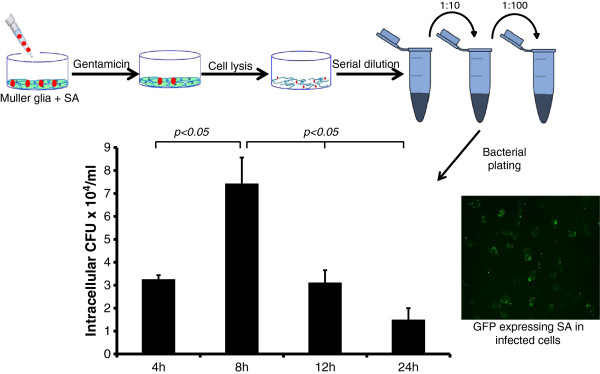
**Müller glia phagocytize *S. aureus *and kill them.** To assess the phagocytic activity of Müller glia, MIO-M1 cells were challenged with *S. aureus* (SA) as described in the ‘Materials and Methods’ section. After two hours of incubation, the cells were washed and kept in fresh medium containing gentamicin (200 μg/ml). At the desired time point, cells were lysed. The release of intracellular bacteria was quantitated via serial dilution and plate count. GFP-expressing SA was used to visualize intracellular bacteria. Note: at an earlier time point (eight hours), more colony forming units (CFUs) were observed, indicating that the number of internalized bacteria was greater in comparison to the later time points, where the CFUs are decreased, indicating the intracellular killing of SA over time. The data represent mean ± SD three independent experiments. Statistical analysis was performed using a student’s *t*-test and the data for various time periods were significantly different from each other (*P < 0.05).

Having demonstrated the bactericidal activity of Müller glia-conditioned media, we next sought to determine whether Müller glia can phagocytize the bacteria (specifically, *S. aureus*), an ability commonly exhibited by innate immune cells. Using a gentamicin protection assay (Figure 8), our data showed that at eight hours, a significantly increased number of bacteria were internalized by Müller glia, indicating phagocytic activity. At the 12-hour time point, the recovery of viable intracellular bacteria dropped below the level at four hours (the time of invasion). This trend continued, with a drastically reduced number of phagocytized bacteria at 24 hours. Fluorescence microscopy results also indicate the phagocytosis of GFP expressing *S. aureus* by Müller glia. Taken together, these results indicate that Müller glia can phagocytize and kill bacteria.

## Discussion

In this study, we demonstrated that, in addition to the inflammatory response, the activated Müller glia exhibit antibacterial responses, as evidenced by the increased expression and production of AMPs (defensins and LL37), ROS, and NO, a phenotype commonly displayed by mucosal epithelial cells. We also elucidated the functional role (that is, the bactericidal activity) of activated Müller glia derived AMPs. Furthermore, Müller glia were also found to kill the bacteria by internalization, a phenomenon exhibited by classical immune cells (polymorphonuclear neutrophils (PMNs) and macrophages). Taken together, our data provide evidence that Müller glia produce various antimicrobials to inhibit bacterial growth, suggesting their critical role in retinal innate defense.

Innate immune cells use several strategies to limit bacterial growth in infected tissues [30]. On mucosal surfaces, these innate immune defenses include the secretion of antibacterial factors, such as antimicrobial peptides [21]. Since the mucosal surfaces are constantly exposed to a wide variety of physical, chemical, and biological insults, the expression of AMPs could be either constitutive or inducible. By contrast, whether or not similar mechanisms operate in the retina, a tissue that is never exposed to the external environment, is largely unexplored. Although clinical evidence does support the presence of AMPs (β-defensins) in human vitreous, albeit at a low level, which retinal cells produce the AMPs in the vitreous is still unknown. In this study, we provide the first evidence that retinal Müller glia contribute towards the production of β-defensins. Furthermore, their expression is inducible in response to bacterial challenge, implicating their role in the antimicrobial defense in the retina. Defensins are a class of particularly abundant and widely distributed AMPs characterized by a cationic, β-sheet rich amphipathic structure stabilized by a conserved three-disulfide motif ranging in size from 29 to 47 amino acids. It was once believed that the expression of β-defensins was limited to epithelial cells. However, several studies have now shown that they can also be secreted by other cell types. For example, Lehman *et al.* have shown the expression of defensin 1 and 2 by kidney cells with chronic bacterial infections. Similarly, Gambichler *et al.* have shown the expression of β-defensins in basal cell carcinoma [31,32]. Our data also revealed that Müller glia have the ability to produce HBD1, HBD2, and HBD3 in response to bacterial (*S. aureus*) infection.

In addition to β-defensins, Müller glia were also found to express and secrete LL37, the only cathelicidin antimicrobial host defense peptide expressed in humans. This observation is consistent with our previous study [29]. The important antimicrobial property of LL37 *in vivo* relates to its potent anti-inflammatory activity and its selective ability to modulate a favorable immune response. For example, LL37 not only kills bacteria, but it also inhibits biofilm formation [33,34], an important virulence factor in the pathogenesis of staphylococcal endophthalmitis [35]. LL37 is also reported to influence many aspects of innate immunity, such as chemotaxis, angiogenesis, and wound healing. However, it is not completely understood whether these activities are associated with certain cell receptors and/or epidermal growth factor receptor (EGFR) [36-38]. Taken together, our study demonstrates that Müller glia produce multiple AMPs in response to bacterial infection. Furthermore, our data show that the secreted AMPs contributes to the observed bactericidal activity of Müller glia conditioned media, as evidenced by loss of this activity by proteinase K treatment (Figure 6). However, further studies are warranted to define the contribution of each individual AMP.

Both the host and the bacteria require iron for metabolism and growth. Thus, the availability of free iron is an important factor in bacterial pathogenesis. The host has evolved mechanisms of ‘withholding’ iron from tissue fluids in an attempt to limit bacterial growth. Systemic iron homeostasis in humans is controlled by the 25 amino acid peptide hormone hepcidin, produced by hepatocytes. The expression of hepcidin has been reported in retinal pigmented epithelial (RPE) cells [39,40], as well as in mouse Müller glia and photoreceptor cells [40]. The induced expression of hepcidin in Müller glia in response to bacterial challenge suggests that hepcidin production during the innate immune response in endophthalmitis may enable the retina to withhold iron from extracellular pathogens such as *S. aureus*. By inhibiting ferroportin function in macrophages and other innate cells, hepcidin reduces iron availability in the vitreous and, by extension, potentially limits growth of the pathogen.

The *in vivo* phagocytic activity of Müller cells was described more than eight decades ago [4,41]. Studies have shown that Müller cells can phagocytize type II collagen and cellular debris from dead cells and latex beads [4,42]. To our knowledge, there is no evidence that these cells can phagocytize pathogens. In this study, we evaluated the phagocytotic properties of Müller cells following *S. aureus* infection. Our data showed that Müller cells can not only phagocytize bacteria, but can kill them intracellularly, as evidenced by reduced bacterial CFUs in two independent experiments (both adherent and suspension cell cultures). In order to understand the mechanism of intracellular killing by Müller glia, we hypothesized that, similar to other innate immune cells (for example, neutrophils), Müller glia may generate ROS and RNS. To this end our data showed that Müller glia produces ROS in response to *S. aureus* challenge. Few reports have linked ROS generation and TLR2/4 signaling [43,44]. Since, our previous study showed that Müller glial innate responses towards *S. aureus* are mediated via TLR-2 [5], we cannot rule out the possible involvement of TLR2-signaling in ROS generation by Müller glia. Moreover, as ROS play an important role in anti-bacterial defense, it is not surprising that signaling from cell-surface TLRs, which predominantly recognize ligands derived from bacteria, induce the generation of ROS. Similar to ROS, NO produced by iNOS is another common component of the host’s innate immune response against a wide variety of pathogens. The killing of a microorganism by NO is quite remarkable, as most of the antimicrobial actions exerted by this diatomic radical against most microorganisms manifest via cytosis [45,46]. NO can also arrest the replication of phylogenetically diverse microorganisms such as *Candida albicans, E. coli, Salmonella enterica,* and *Burkholderia pseudomallei*[46-49]. In the current study, we showed that Müller glia can produce NO following *S. aureus* infection, implicating its role in bacterial killing during ocular infections.

Although, our data showed that Müller glia produce ROS and NO in response to bacterial challenge, it should be noted that these reactive oxidant species are involved in multiple complex interactions between the invading pathogen and the host [50]. During microbial infection, the pathogen produces these oxidants to enable them to have a survival advantage in the host environment. On the other hand, the host cells, especially the phagocytes, produce them as a counteractive mechanism to kill pathogens. However, if this vicious cycle continues it may lead to inflammatory tissue damage in the retina. Thus, the host cells including Müller glia have evolved complex adaptive mechanisms to deflect oxidant-mediated damage, including enzymatic and nonenzymatic oxidant-scavenging systems [51]. Furthermore, the Müller glia release neurotrophic factors, they uptake and degrade excitotoxins, glutamate, and secrete glutathione (a potent antioxidant) to protect retinal neurons [52].

## Conclusions

**Figure 9:**
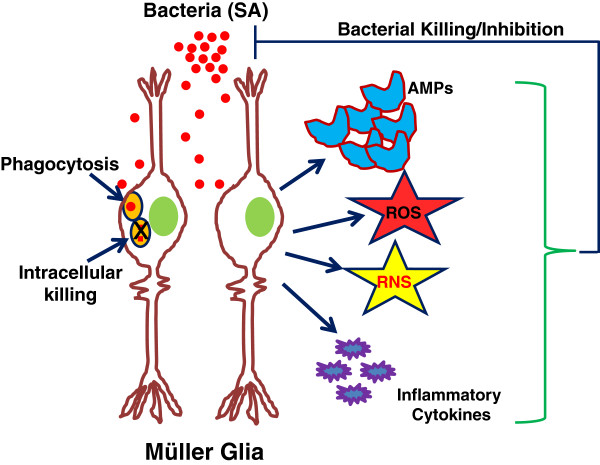
**The mechanisms of the Müller glial antimicrobial response.** Following microbial (bacteria) insult, Müller glia elicit innate defense responses in an attempt to limit bacterial growth. These include the production of antimicrobial peptides (AMPs), reactive oxygen species (ROS), and reactive nitrogen species (RNS), which possess the ability to kill the bacteria directly. Additionally, Müller glia can phagocytize and kill the pathogens intracellularly.

To conclude, our study suggests that in addition to serving as a supportive cell, retinal Müller glia actively participate in the retinal innate defense against microbial pathogens. Müller glia directly contribute to bacterial killing via the production of AMPs (for example, defensins, LL37), the generation of ROS/RNS, and phagocytosis (Figure 9). In view of the alarming increase in antibiotic resistance, the endogenous production of antimicrobial molecules exerting additive or synergistic effects could be a novel approach to the prevention of ocular infections such as endophthalmitis. Thus, understanding the processes and mechanisms by which the innate immune system operates in the retina will help in developing new therapies that will not only treat retinal disease, but could also aid in the prevention of retinal disease.

## Abbreviations

AMP: antimicrobial peptide; HBD: human β defensin; ROS: reactive oxygen species, RNS, reactive nitogen species; DCFH-DA: dichloro-dihydro-fluorescein diacetate; TLRs: toll like receptors; ILM: inner limiting membrane; PRRs: pattern recognition receptors; PAMPs: pathogen-associated molecular patterns; BRB: blood retinal barrier; SA: *Staphylococcus aureus*; CFUs: colony forming units; MOI: multiplicity of infection; GFP: green fluorescent protein; CAMP: cathelicidin antimicrobial peptide; MPO: myeloperoxidase; NOD2: nucleotide binding oligomerization domain containing protein 2; NLRP3: NOD like receptor protein 3; MAPK: mitogen activated protein kinase; iNOS: inducible nitric oxide synthase; HBSS: Hank’s balanced salt solution; PMNs: polymorphonuclear neutrophils.

## Competing interests

All authors declare that they have no competing interests.

## Authors’ contributions

PKS designed and performed the experiments, analyzed the data and wrote the manuscript. MJS performed the phagocytosis assay and contributed to manuscript proof reading. AK contributed in directing and planning the course of the study as well as manuscript writing. All authors read and approved the final version of the manuscript.
